# Association of Frailty With Clinical Outcomes in Patients Receiving Primary Prevention Implantable Cardioverter Defibrillators: A Prospective Cohort Study

**DOI:** 10.1111/anec.70061

**Published:** 2025-03-14

**Authors:** Dmitry Neymark, Christopher Lee, William F. McIntyre, Maria Higgins, James W. Tam, Colette Seifer

**Affiliations:** ^1^ Section of Cardiology, Department of Internal Medicine University of Manitoba Winnipeg Manitoba Canada; ^2^ Division of Cardiology University of Calgary Calgary Alberta Canada; ^3^ Section of Cardiology, Department of Internal Medicine McMaster University Hamilton Ontario Canada; ^4^ Section of Palliative Care, Department of Internal Medicine University of Manitoba Winnipeg Manitoba Canada

**Keywords:** cardiac resynchronization therapy, cardiovascular disease, CRT, CVD, frailty assessment, ICD, implantable cardioverter defibrillator, primary prevention, SCD, sudden cardiac death

## Abstract

**Background:**

Frailty predisposes individuals to morbidity and mortality. Increasing numbers of elderly and comorbid individuals are undergoing primary prevention implantable cardioverter defibrillator (ICD) device placement. Little is known about the association of frailty with post‐device implantation outcomes.

**Methods:**

We conducted a single‐center, prospective cohort study of 71 patients who underwent primary prevention ICD insertion and who had their baseline frailty status assessed using the Fried index. Participants were followed for a median period of 7.8 years.

**Results:**

The mean age (± SD) was 70.6 ± 4.5 years. 12 (17%) patients met the criteria for frailty. 23 (33%) patients received cardiac resynchronization therapy. Frailty was associated with a significantly higher incidence of mortality (HR [95% CI]; 3.9 [1.2–12.1]), ED visits (2.7 [1.1–6.7]), and hospitalizations (2.8 [1.1–7.6]). Within the non‐frail cohort, there was no association between Fried frailty scores and adverse outcomes. None of the frail patients received appropriate shock therapy.

**Conclusion:**

Among primary prevention ICD recipients, frailty is associated with worse mortality and morbidity. Clinicians should consider frailty when discussing risks and benefits with this patient population.

## Graphical abstract

Frailty assessed via the Fried Frailty Index is associated with greater mortality, incidence of hospitalizations, and emergency room visits.

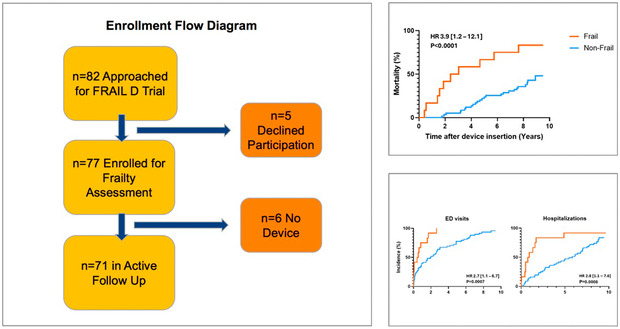

## 1 Introduction

Frailty is defined as the clinical syndrome marked by decreased physiological reserve and vulnerability to stressors (Afilalo et al. 2009; Clegg et al. 2013). An established association exists between frailty, advanced age, and chronic illness, including cardiovascular disease (CVD) (Fried et al. 2001). The diagnoses of coronary artery disease and heart failure are associated with a several‐fold increase in the prevalence of frailty among elderly patients and vice versa (Afilalo et al. 2009). Among patients with known CVD, those who are frail appear to face an elevated risk of mortality compared to non‐frail individuals (Afilalo et al. 2009; Clegg et al. 2013). In the peri‐procedural setting, frailty status is associated with higher rates of complications and prolonged recovery (Dasgupta et al. 2009; Chen et al. 2019).

Implantable cardioverter defibrillators (ICDs) and cardiac resynchronization therapy defibrillators (CRT‐D) are used for the prevention of sudden cardiac death. Their use in elderly and comorbid patients remains an area of controversy due to concerns about the elevated risk of complications and diminished benefit in the context of increased mortality from competing non‐cardiac causes (Bibas et al. 2016; Steinberg et al. 2014; Vijayarajan et al. 2024; Akta ş et al. 2023). A recent *post hoc* review of the SCD‐HeFT trial by Segar et al. (2024) pointed to the loss of the mortality benefit of ICD therapy among patients with a high frailty burden. Conversely, there is indirect evidence pointing to a potential survival benefit with implantable defibrillator placement in elderly patients over the age of 75, and the current consensus points to a relatively low risk of peri‐procedural complications, thus making it a potentially appealing intervention even in the most frail (Bibas et al. 2016; Kong et al. 2011).

To date, our understanding of frailty among patients receiving implantable device therapies is largely derived from subgroup analyses of studies that stratified individuals based on age or number of comorbidities (3). Neither of the above represents comprehensive markers of physiological reserve, and more work is needed to adequately elucidate the impact of frailty on patient outcomes following ICD and CRT‐D device placement. The objective of this study is to estimate the association of baseline frailty with mortality and morbidity in patients who receive primary prevention ICD and CRT‐D devices.

## 2 Methods

### 2.1 Study Design and Setting

This was a single‐center, prospective cohort study conducted in Winnipeg, Manitoba, Canada, at a tertiary care cardiac center with a catchment area of 1.4 million and 260 new implants per year. The institutional review board at the University of Manitoba approved the trial protocol (H2014:290; H2024:185). All participants provided informed written consent at the time of enrollment.

### 2.2 Study Participants and Enrollment

Participants were recruited consecutively between November 25, 2014, and January 2, 2017. Eligible patients were male and female, age 65 and older, referred to the Electrophysiology Service, and who underwent primary prevention ICD/CRT‐D implantation. Primary prevention was defined as patients deemed at higher risk for sudden cardiac arrhythmic death but have not yet sustained a ventricular arrhythmia. Patients with pre‐existing devices were excluded from the study.

### 2.3 Definition of Frailty

Frailty was assessed in person at the ICD clinic via the Fried frailty index by a trained clinic nurse (Fried et al. 2001) (Table 1). The assessment time was 10–15 min per patient.

**TABLE 1:** Fried's frailty index^a^ and baseline prevalence of functional impairements in a cohort of primary prevention ICD Recipients.

Domain	Criterion	*n* (%)
Slowness	5‐m gait speed test scoring > 6 s.	16 (23%)
Weakness	Grip strength of < 30 kg and < 20 kg for men and women respectively.	11 (15%)
Physical activity	Activity < 383 and < 270 kcal/week for men and women respectively as determined by Physical Activity Scale for the Elderly (PASE) questionnaire	8 (11%)
Exhaustion	Self‐reported	31 (44%)
Weight loss	Self‐reported	26 (37%)

^a^ Frailty is defined by the presence of impairments in 3 or more of the above domains.

Patients who had impairments in 3 or more of the measured domains were defined as frail. Participants that did not meet the criteria for frailty were further sub‐stratified according to the number of measured domains with impairments—0 (robust), 1, or 2 (intermediate/pre‐frail).

### 2.4 Outcomes

All‐cause mortality was the primary outcome evaluated in the study. Secondary outcomes included emergency department presentations, hospitalizations, heart failure exacerbations, ICD shocks, and deactivation of shock therapies.

### 2.5 Follow‐Up

Patients underwent in‐person follow‐up assessments at 1,6,12, and 24 months post‐device insertion. In‐person assessments took place between November 25, 2014, and January 2, 2019. Further follow‐up was done through province‐wide electronic patient medical records.

### 2.6 Statistical Analysis

Baseline characteristics were compared between groups using an independent‐sample *t*‐test for continuous variables and Chi‐square or Fisher exact tests for categorical variables. In cases of skewed distributions, median, interquartile ranges, and the Mann–Whitney *U*‐test for continuous variables were applied. Cumulative incidence of the outcomes of interest was compared via Kaplan–Meier curves and the log‐rank test. Relative incidences of outcomes of interest were expressed in the form of unadjusted hazard ratios.

Statistical analyses were performed using appropriate software (Prism Version 10.3.0) All statistical tests were two‐sided. P‐values less than 0.05 were considered significant.

## 3 Results

Eighty‐two consecutive patients were assessed for eligibility in the study between November 2014 and January 2017. The median follow‐up period was 7.8 years. Five patients declined participation in the study, and another six patients declined device placement. The remaining 71 (87%) patients were included in the final analysis.

### 3.1 Baseline Characteristics

Baseline demographic and medical characteristics of the frail (*n* = 12) and non‐frail (*n* = 59) patient cohorts are depicted below (Tables 1 and 2). The mean age (± SD) was 70.6 ± 4.5 years; 22% of the participants were female. Twelve patients (17%) met the criteria for frailty. Among non‐frail patients, 22 (35%), 22 (31%), and 12 (17%) patients exhibited functional impairments in zero, 1, and 2 functional domains, respectively. Twenty‐three (33%) patients underwent CRT‐D insertion; ICD was implanted in 48 (67%) patients, four of whom underwent a subsequent upgrade from ICD to CRT‐D. Frail patients exhibited a higher incidence of coronary artery disease, lower creatinine clearance, greater prevalence of NYHA 3 symptoms at baseline, and a higher incidence of prescriptions for nitrates and loop diuretics.

**TABLE 2:** Baseline characteristics by frailty status.

	Frail (12)	Non‐frail (59)	*p*
Age	71.8 ± 6.1	70.4 ± 4.2	0.314
Female	8%	26%	0.183
BMI	28.8 ± 6.5	31.1 ± 5.4	0.201
ICD^a^	75%	61%	0.7
CRT‐D^b^	25%	39%	0.7
Non‐ischemic cardiomyopathy (%)	17%	43%	0.087
Ejection fraction (%)	26% ± 6%	27% ± 7%	0.480
Coronary artery disease	92%	60%	**0.034**
Diabetes	75%	46%	0.067
Smoker	0%	8%	0.323
Hypertension	75%	54%	0.180
Dyslipidemia	33%	39%	0.720
Atrial fibrillation	58%	35%%	0.135
Creatinine	130 ± 36	101 ± 34	**0.013**
eGFR	52 ± 19	75 ± 34	**0.023**
NYHA 1^c^	8%	17%	0.437
NYHA 2	17%	40%	0.084
NYHA 3	75%	43%	**0.045**
*Baseline medications*			
Aspirin	75%	69%	0.689
Dual antiplatelet therapy	33%	17%	0.198
Statin	67%	72%	0.727
Nitrates	33%	11%	**0.045**
Ace inhibitor or ARB^d^	92%	83%	0.433
MRA^e^	42%	31%	0.450
Loop diuretic	100%	65%	**< 0.0001**
Calcium channel blocker	25%	9%	0.113
Beta blockers	92%	82%	0.375
Amiodarone	17%	8%	0.308
Digoxin	8%	8%	0.972
Oral anticoagulation	50%	25%	0.075

*Note:* Statistical Significance ‐ *p* < 0.05.

^a^ Implantable cardiac defibrillator.

^b^ Cardiac resynchronization therapy.

^c^ New York heart association.

^d^ Angiotensin receptor blocker.

^e^ Mineralocorticoid receptor antagonist.

### 3.2 Primary Outcomes

**FIGURE 1:**
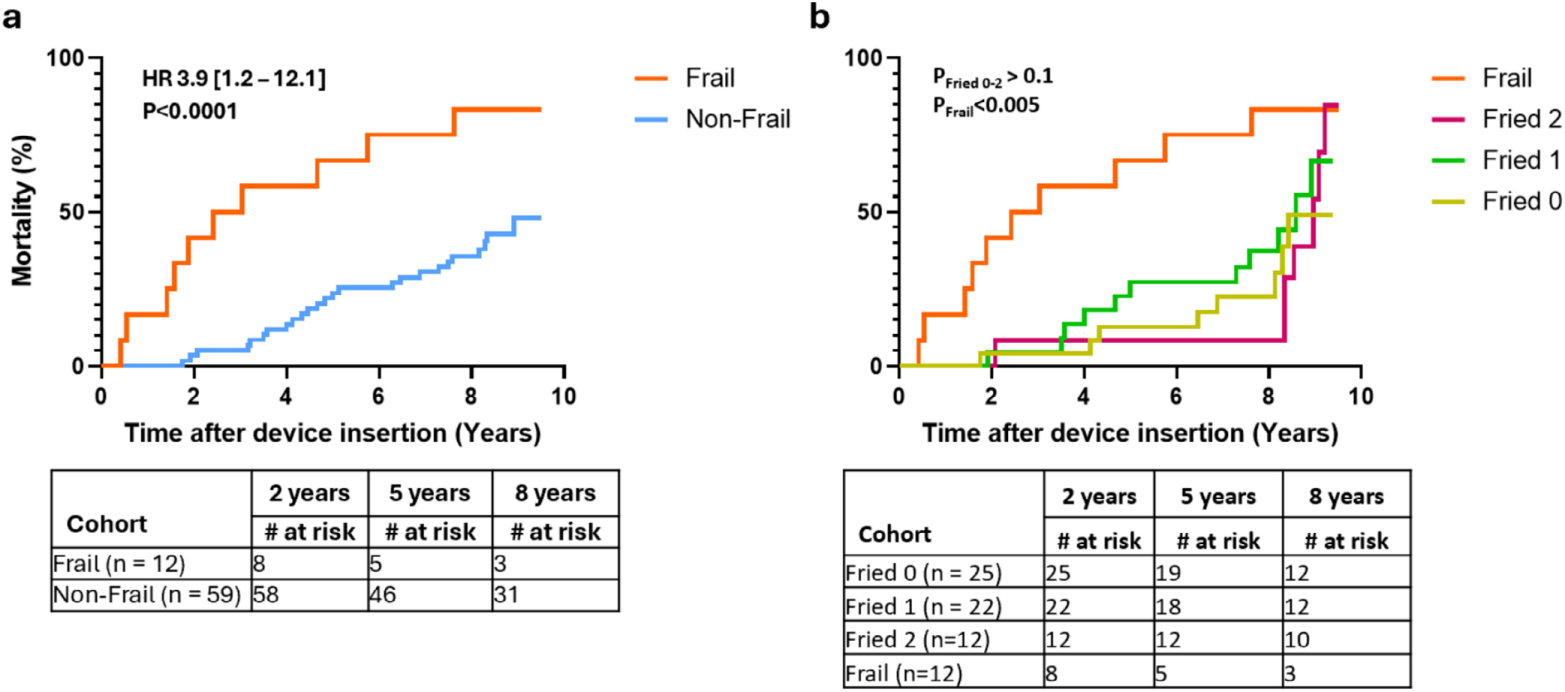
Kaplan–Meier estimates of all‐cause mortality following implantable cardioverter defibrillator (ICD) or cardiac resynchronization therapy defibrillator (CRT‐D) implantation stratified by baseline Frailty status (a) and precise number of functional impairments (Fried 0,1,2) measured via Fried's frailty scale (b). HR—Hazard ratio. *p*‐values are obtained via log‐rank test; P_Fried 0–2_—Aggregate P‐values among Fried 0–2 subgroups, P_Frail_—Aggregate P‐values comparing Frail and Fried 0–2 subgroups.

Frail patients had significantly (*p* < 0.001) higher mortality (83% vs. 42%), HR [95% CI]; 3.9 [1.2–12.1] across the entire follow‐up period, with a median survival of 2.7 years compared to the non‐frail patients whose median survival extended beyond the 7.8‐year follow‐up period (Figure 1a).

Further sub‐stratification of the non‐frail patient cohort according to the number of domains of frailty revealed no significant differences in 8‐year mortality rates (%) and median survival times (years) among patients who have met 0 (30%; > 9.0), 1 (38%; 8.6), or 2 (30%; 9.0) criteria for frailty. All three patient groups exhibited significantly (*p* < 0.005) lower mortality rates and longer survival times compared to the frail patients (Figure 1b).

### 3.3 Secondary Outcomes

#### 3.3.1 ED Visits

**FIGURE 2:**
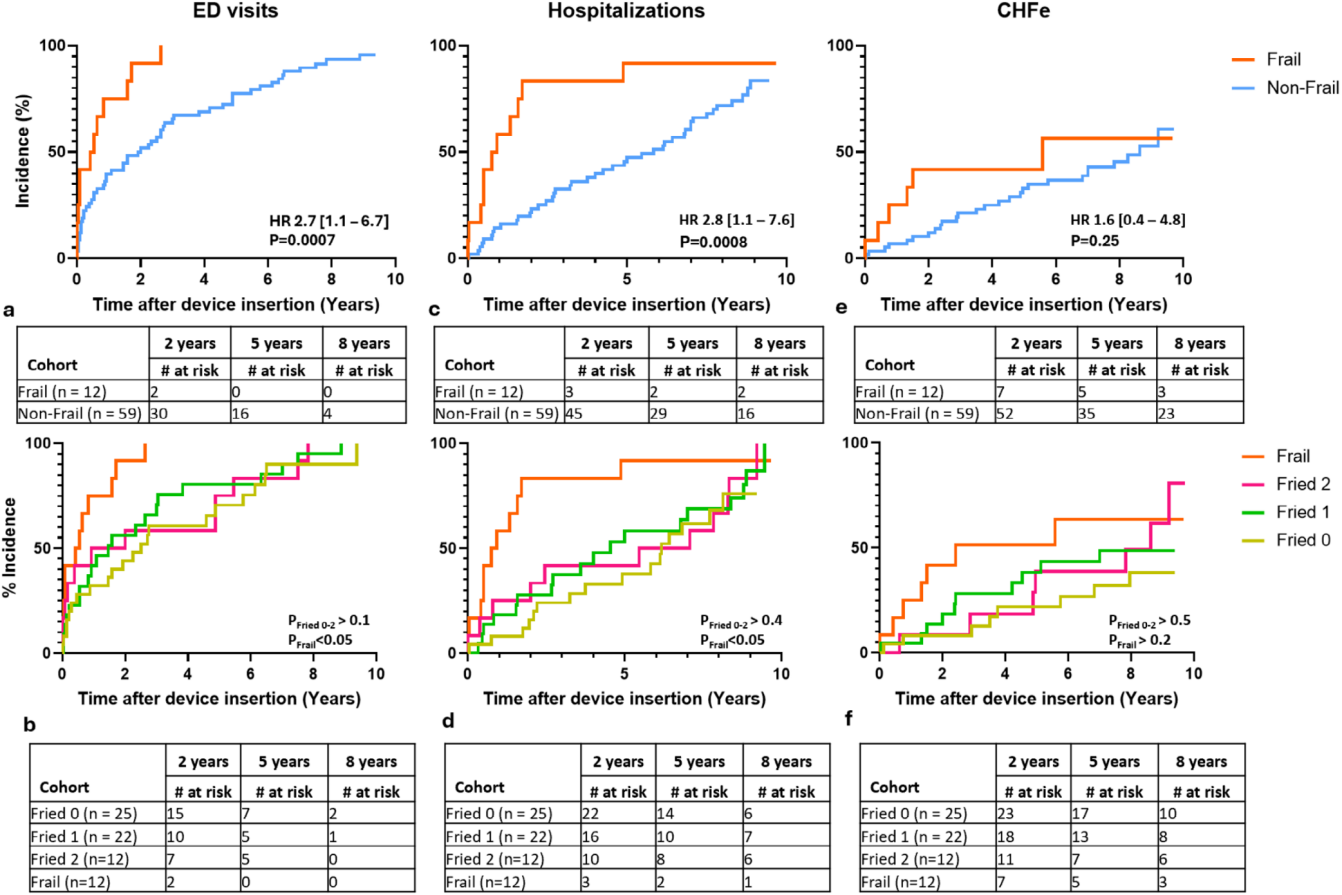
Kaplan–Meier estimates of incidences of ED visits (a, b); hospitalizations (c, d); and heart failure exacerbations (e, f) following ICD or CRT‐D implantation. Outcomes were stratified by baseline Frailty status (top row—a, c, e) and exact number of functional impairments (Fried 0, 1, 2) measured via Fried's frailty scale (bottom row—b, d, f).

Frail patients had a higher and earlier incidence of ED visits (100% vs. 93% HR 2.7 [1.1–6.7]) over the course of the follow‐up period (Figure 2a). Median time to the first ED visit was significantly (*p* < 0.001) shorter among frail (0.48 years) compared to the non‐frail (2.0 years) patients. The two cohorts exhibited a similar mean number of ED visits (Frail 7.9 ± 12.7 visits/person vs. Non‐frail −6.0 ± 10.6 visits/person).

The three non‐frail subgroups demonstrated similar median times to the first presentation to ED—2.5, 1.5, and 1.5 years for those with 0, 1, and 2 functional impairments, respectively. The above incident‐free survival periods were significantly longer compared to the frail (*p* < 0.05) cohort (Figure 2b). Additionally, patients with 0, 1, and 2 functional impairments had a similar number of mean visits—3.5 ± 4.2; 4.1 ± 6.0; 11.3 ± 20.8 visits/person, respectively.

#### 3.3.2 Hospitalizations

Incidence of hospital admissions was higher among frail patients compared to the non‐frail cohort (93% vs. 73% HR 2.8 [1.1–7.6]) (Figure 2c). The median time of first hospitalization was 0.8 years post‐device insertion for frail patients and 5.8 years among non‐frail participants. The mean number of days spent in the hospital was similar across both patient cohorts, not accounting for the differences in survival (frail −29.3 + 46.2 days/person; non‐frail −22.8 ± 32.2 days/person).

Patients with 0 (6.2 years), 1 (4.5 years) and 2 (6.3 years) functional impairments exhibited similar median times to first hospitalization (Figure 2d), all of which were significantly longer compared to frail patients (*p* < 0.05). Mean duration of hospitalization was also similar across the three subgroups—14.3 ± 15.5; 29.08 ± 35.5; 26.6 ± 45.5 days/person, respectively.

#### 3.3.3 CHF Exacerbations

No significant differences in the incidence or time to the initial episode (Frail—6.6 years; Non‐frail—8.6 years) of CHF exacerbations (HR 1.6 [0.6–4.8]) were observed among patient cohorts (Figure 2e). Similarly, stratification of the non‐frail patients according to the number of functional impairments did not reveal any differences in incidences of CHF exacerbations (Figure 2f).

#### 3.3.4 ICD Shocks

No frail patients received an appropriate shock over the course of the study (Table 2). Eleven non‐frail patients (28%) received an appropriate shock during the study, with a mean of 2 ± 1.4 shocks per participant shocked. One frail (9%) and two non‐frail (3%) were shocked inappropriately.

#### 3.3.5 Deactivation of Therapies

Three frail (25%) and 10 non‐frail (17%) patients underwent deactivation of tachyarrhythmia therapies.

## 4 Discussion

We analyzed the relationship between frailty and post‐ICD insertion mortality and morbidity. The main finding of our study is that baseline frailty predicts increased mortality following an implantable device insertion over the course of the 8‐year follow‐up period. Additionally, frail patients exhibited greater morbidity as marked by higher rates of ED visits and hospitalizations. Interestingly, further exploration of patients according to the number of frailty domains with measured impairments pointed to a threshold relationship between the number of impairments and adverse clinical outcomes. In other words, the presence of functional impairments did not predict morbidity and mortality until the critical point of at least three compromised domains was reached.

Our results are consistent with the database analyses by Green et al. (Green et al. 2016) and Mohamed et al. (Mohamed et al. 2019) that found frailty to be an independent risk factor for post‐ICD implantation mortality. Although studies examining age and the number of comorbidities as indirect measures of low physiologic reserve also reported greater mortality rates post‐device insertion (Steinberg et al. 2014; Vijayarajan et al. 2024; Lee et al. 2007; Kubala et al. 2017). Our findings of similar outcomes among robust and pre‐frail phenotypes contrast Fried's and colleagues' (Fried et al. 2001) original findings of the dose–response relationship between the number of frailty components and mortality. Given the small sample size across the subgroups in our study, the discrepancy is likely to represent the lack of statistical power to detect more subtle differences in outcomes among the non‐frail subtypes.

Importantly, none of the frail patients received an appropriate shock over the course of the study. Three (25%) frail patients chose to have shock therapies deactivated, with one additional frail patient requesting a deactivation of therapies but being unable to attend an in‐person appointment due to frailty and geographic distance. While the interpretation of the above findings is limited by a small sample size, the lack of observable therapeutic benefit supports the concerns regarding the diminished utility of ICD therapies in patients who are at higher risk of non‐arrhythmic death from competing causes or deterioration to the point of not benefiting from resuscitative care (Bibas et al. 2016; Steinberg et al. 2014; Vijayarajan et al. 2024; Lee et al. 2007).

While frailty as defined by the Fried index appears to have the capacity to serve as a marker for increased risk of mortality post‐device insertion, at this time the utility of implementing formal frailty assessments in assessing peri‐procedural risk remains unclear. Nonetheless, from a practical standpoint, the frailty assessments were completed within approximately 10–15 min, making them a potentially feasible component of pre‐procedural evaluation.

Current guidelines on the implementation of ICD suggest a holistic approach to determining the appropriateness of implantable device therapy (Bennett et al. 2017). Frailty scores have been described as an imperfect but a potentially useful tool in identifying individuals who were less likely to benefit from ICD therapies, and more importantly, developing a comprehensive approach toward understanding patients' care trajectory (Rowe et al. 2014).

### 4.1 Strengths and Limitations

To our knowledge, this is the first prospective study evaluating the prevalence and clinical outcomes of frailty in a Canadian population referred for primary prevention ICD. The present study examined frailty as a distinct clinical phenotype using standardized and validated criteria (Fried et al. 2001). Furthermore, we assessed subclinical frailty and its association with adverse postprocedural outcomes.

There are several limitations to the present study. It is a single‐center patient cohort, which limits its generalizability and statistical power. From a design standpoint, the study did not include a control group of patients who did not undergo an ICD or CRT‐D placement. As such, we cannot make inferences about the impact of device insertion on participants. The present study did not formally assess the rates of device complications. Our study did not capture the cause of mortality for deceased individuals, which would have been helpful in evaluating mortality from competing causes (Bibas et al. 2016; Steinberg et al. 2014; Vijayarajan et al. 2024; Akta ş et al. 2023). Finally, we did not perform multivariate analysis to compute adjusted odds ratios for frailty in predicting adverse outcomes due to the small sample size. As such, our results do not describe frailty as an independent predictor of morbidity and mortality.

## 5 Conclusions

The present single‐center cohort trial found frailty, as defined by Fried's frailty index, to be a predictor of mortality, increased incidence of ED visits, and hospitalizations following ICD or CRT‐D insertion. Frail patients did not receive appropriate ICD shocks over the course of the follow‐up period. Our findings point to the attenuated benefit of implantable device therapies in Frail individuals. Evaluating the role of formal frailty assessments in peri‐procedural planning represents an important area of further research.

## Author Contributions

D.N.: data collection, data analysis, manuscript writing. C.L.: study design, data collection, data analysis, manuscript writing. W.F.M.: study design, data analysis, manuscript writing. M.H.: data collection. J.W.T.: study design, data analysis. C.S.: study design, data collection, data analysis, manuscript writing.

## Disclosure

W.F.M.: speaking iRhythm, consulting AtriCure.

## Consent

The authors confirm that patient consent forms have been obtained for this article.

## Conflicts of Interest

The authors declare no conflicts of interest.

## Data Availability

The data that support the findings of this study are available from the corresponding author upon reasonable request.
